# Prevention and treatment of rheumatoid arthritis through traditional Chinese medicine: role of the gut microbiota

**DOI:** 10.3389/fimmu.2023.1233994

**Published:** 2023-09-14

**Authors:** Yujiao Liang, Mengyao Liu, Yingxue Cheng, Xinchang Wang, Weijie Wang

**Affiliations:** ^1^ The Second Clinical Medical College, Zhejiang Chinese Medical University, Hangzhou, China; ^2^ School of Basic Medical Science, Zhejiang Chinese Medical University, Hangzhou, China; ^3^ Department of Rheumatology, the Second Affiliated Hospital of Zhejiang Chinese Medical University, Hangzhou, China; ^4^ Institute of Basic Theory for Chinese Medicine, China Academy of Chinese Medical Science, Beijing, China

**Keywords:** Gut Microbiota, Traditional Chinese Medicine, Rheumatoid Arthritis, immunology, rheumatic and immune disease

## Abstract

Recently, despite the increasing availability of treatments for Rheumatoid arthritis (RA), the incidence of RA and associated disability-adjusted life years have been on the rise globally in the late decades. At present, accumulating evidence has been advanced that RA is related to the gut microbiota, therefore, the therapeutic approaches for RA by regulating the gut microbiota are anticipated to become a new means of treatment. Traditional Chinese medicine (TCM) can regulate immunity, reduce inflammation and improve quality of life in various ways. Moreover, it can treat diseases by affecting the gut microbiota, which is a good way to treat RA. In this review, we mainly explore the relationship between TCM and gut microbiota regarding the perspective of treating RA. Moreover, we comprehensively summarize the roles of gut microbiota in the onset, development, progression, and prognosis of RA. Additionally, we elucidate the mechanism of TCM prevention and treatment of RA by the role of microbiota. Finally, we provide an evidence-based rationale for further investigation of microbiota-targeted intervention by TCM.

## 1 Introduction

RA is an immune-mediated disease, which is characterized by multi-joint, symmetrical, and invasive changes in small joints. RA usually occurs with symmetrical swelling and pain in multiple joints like hands, wrists, and feet. Morning stiffness, joint pain and tenderness, and joint swelling can be seen in the early time, and joint deformity occurs in patients in the later stage. In addition to the articular manifestations, there will also be some extra-articular manifestations such as cutaneous rheumatoid nodules, rheumatoid vasculitis, etc. Cardiovascular diseases and respiratory diseases are the most usual complications of RA patients and the common causes of death in RA patients (1). The cause and pathogenesis of RA are complicated. Genetic susceptibility and environmental factors are the basis for RA’s pathogenesis and development (2). Growing evidence has indicated that changes in gut microbiota are correlated to RA. Gut microbiota disturbance can be discovered in RA patients, and healthy microbiota can be partially recovered after disease-modifying antirheumatic drugs (DMARDs) treatment (3). In addition, the imbalance of certain bacterial lineages and changes in the metabolism of gut microbiota lead to changes in the host immune spectrum, leading to the pathogenesis of RA (4).

Traditional Chinese medicine (TCM) has been used for more than 2000 years in China with its unique theories (5, 6). Some TCM monomers and prescriptions have been used clinically to treat RA. TCM can treat RA in various ways such as immune network regulation and inhibition of inflammatory factors. In addition, TCM can also treat RA by regulating gut microbiota. This review will explore the mechanism and the progress of studies of TCM in treating RA by regulating gut microbiota, and provide new clues in the treatment of RA.

## 2 Advantages of Traditional Chinese Medicine in treating Rheumatoid Arthritis

RA is characterized by systemic damage and inflammation, affecting joints and extra-articular organs, which can lead to serious injury of joints and disability. Conventional treatments for RA encompass non-steroidal anti-inflammatory drugs (NSAIDs), immunosuppressive agents, glucocorticoids, and DMARDs. These drugs can effectively inhibit inflammation. However, long-term use may induce some adverse reactions, such as cardiovascular and gastrointestinal side effects, osteoporosis, etc (7). Fortunately, the types of drugs used in treating RA have steadily increased. The emergence of biological preparation like TNF-a, IL-6, and a small molecule targeted drugs such as JAK can effectively inhibit the progression of RA. But long-term use of biologics also leads to side effects that increase the risk of severe infection (8, 9). Furthermore, RA patients also suffered from great costs of the treatments. A recent meta-analysis estimated an annual direct medical cost in the US for RA of $12,509 for all patients using any treatment regimen and $36,053 for biologics users (10).

The widespread use of DMARD treatment is still hampered by the associated high cost and frequent side effects such as liver damage, cell reduction or increased frequency of infection, and certain cancers (7). Because of the shortcomings of these treatments, there is an active demand to find new drugs with fewer side effects and low costs to treat RA. TCM is rich in a great many chemical constitutions, which not only contain alkaloids, polysaccharides, glycosides, tannins, enzymes, and other active ingredients with therapeutic value but also contain a lot of nutritional active substances (11). TCM acts as an irreplaceable role in the treatment of RA. Some chemical components have been used to treat RA (12). For example, Sinomenine (SIN) has been found to have anti-rheumatic effects and has been ratified by the China Food and Drug Administration (CFDA) for RA treatment (13). The SIN group had a better effect on reducing hypoxia-inducible factor-1α (HIF-1α) and vascular endothelial growth factor (VEGF) than the control group (14). Furthermore, some Chinese patent medicines are widely used in clinical treatment. The Zushima tablet (ZT) has been used to treat RA for ten years with good results (15).

Firstly, the combination of TCM and conventional treatments can enhance the therapeutic effect. In a randomized controlled clinical trial (RCT) of 22 RA patients, it was found that the clinical indicators of the Huayu Qiangshen Tongbi formula plus methotrexate (MTX) group improved earlier than those of the leflunomide (LEF) plus MTX group. In addition, bacterial purine degradation decreased, amino acid biosynthesis increased, and 11 and 9 metabolism pathways changed remarkably with time, higher than the 4 and 2 metabolism pathways in LEF plus MTX group (16). Secondly, TCM can reduce the side effects of conventional treatments. In another RCT comparing Tripterygium wilfordii Hook F (TwHF) with MTX in the treatment of RA, adverse events occurred in 49.3% of patients treated with combination therapy, which was much lower than 62.3% of patients treated with MTX alone (17).

## 3 Relationship between Gut Microbiota and Traditional Chinese Medicine

Gut microbiota is a collection of microorganisms colonized in the intestine (18). It is composed of more than 35,000 bacteria (19). The balance of gut microbiota has the effect of TCM on maintaining immune homeostasis. Both microbial components and microbial metabolites can affect immune regulation. Gut microbiota can affect the response mediated by the pattern recognition receptor encoded by the germ line. The activation of the PRR signaling pathway can lead to the production of antimicrobial peptides, cytokines, etc, and the destruction or change of the signaling pathway can lead to the occurrence of diseases. Monolayer epithelial cells enable microbial metabolites to obtain contact and interaction with host cells, thereby affecting immune response and disease risk (20).Gut microbiota imbalance is the basis of the occurrence and development of many diseases (21). In recent years, it has been indicated that gut microbiota dysbiosis is associated with the development of multiple chronic inflammatory joint diseases, including RA. It may trigger the host’s innate immune system and activate the “gut–joint axis”, which exacerbates the RA (22).

In a cohort study, *Turicibacter* and some *lactobacillus* species were selectively abundant in the gut microbiota of patients with systemic lupus erythematosus (SLE). Transfer of the fecal microbiota to sterile C57/B6 mice resulted in a series of lupus-like manifestations in the mice, and changes in the amino acid metabolism of the microbiota were observed in SLE mice (23). Moreover, compared with the healthy control group, the gut microbiota of Sjogren’s syndrome (SS) patients were enriched in *Lactobacillus salivary*, *Bacteroides fragilis*, and *Ruminococcus gnavus* (24).

TCM can reshape the functional components of herbs through trillions of gut microbiota and enzyme activities secreted by host cells (25). TCM has an impact on the gut microbiota, like regulating the structure and proportion of microorganisms (**Supplementary Table 1**). For example, rhein can reduce uric acid levels by increasing the level of lactobacillus in the intestine of mice (26). The extract of berberine can regulate the gut microbiota of Sprague-Dawley (SD) rats by increasing probiotics like lactic acid bacteria and reducing potential pernicious bacteria such as myxospira (27). Additionally, gut microbiota affects the absorption and metabolism of TCM, which can improve efficacy, reduce toxicity or produce toxic metabolites (28). First of all, gut microbiota can absorb and metabolize TCM. For example, Ellagitanin-containing foods such as strawberries, raspberries, etc, release ellagic acid in the jejunum and metabolize ellagic acid in the gut microbiota, and the resulting metabolites are absorbed (29). Secondly, the lipophilicity of TCM extracts is poor, and the bioavailability is low. The gut microbiota can change its lipophilicity and improve oral bioavailability (30). Therefore, through the role of gut microbiota, the efficacy of TCM may be improved. For instance, mulberry leaves can promote the increase of butyric acid content in the intestine by promoting the multiplication of Prevotella (31). Thirdly, the toxicity of TCM may be reduced. For example, the highly toxic alkaloid diester diterpenoid alkaloids extracted from the roots of Aconitum Carmichael are metabolized by gut microbiota to produce lipid alkaloids or lipo aconitine, and the toxicity is also significantly reduced (32). Finally, toxic metabolites may be produced. Bacteria such as Desulfovibrio can produce high levels of hydrogen sulfide, resulting in loss of colonic epithelial cells and loss of intestinal barrier integrity (33). The compatibility of licorice-kansui in TCM can significantly increase the proportion of Desulfovibrio and increase the concentration of sulfide in feces (34).

Besides, TCM acts on metabolic products of gut microbiota. TCM can regulate the production of Short-chain fatty acids (SCFAs) by acting on the gut microbiota, thus affecting the disease. SCFAs are the main metabolites generated by dietary fiber bacteria during gastrointestinal fermentation (35). The most common are acetic acid, propionic acid, and butyric acid (36). For example, the combined use of licorice (RG) and Beijing Euphorbia (REP) can increase the abundance of *Akkermansia* and *Butyricimonas*, while reducing the content of butyric acid in feces, resulting in adverse reactions to kidney, heart, etc (37). Acetic acid, butyric acid, propionic acid, caproic acid, isobutyric acid, and valeric acid were significantly increased in feces of Collagen-induced arthritis (CIA) rats, while the acetic acid, butyric acid, propionic acid, caproic acid, and valeric acid were significantly decreased after treatment with Angelica Sini Decoction, Among the 6 kinds of gut microbiota improved by Angelica Sini decoction, *g_norank_f_eubacterium_coprostanoligenes_group, g_Romboutsia*, and *g_Lactobacillus* are considered to be the key flora in the treatment of RA by Angelica Sini Decoction (38). Propionic acid and butyric acid can promote the ability of dendritic cells to transform naive T cells into FoxP3 ^+^ Treg by inducing IDO1 and Aldh1A2, and also inhibit the ability of naive T cells to convert into IFN-γ ^+^ T cells (39). Acetic acid and propionic acid can increase the differentiation of naive T cells into Th17 cells in a dose-dependent manner and the derivation of Th1 cells with the presence of IL-12 (40). In summary, SCFAs can regulate T cells through a variety of pathways, thereby affecting intestinal immunity.

## 4 The relationship between Rheumatoid Arthritis and Gut Microbiota

**Figure 1:**
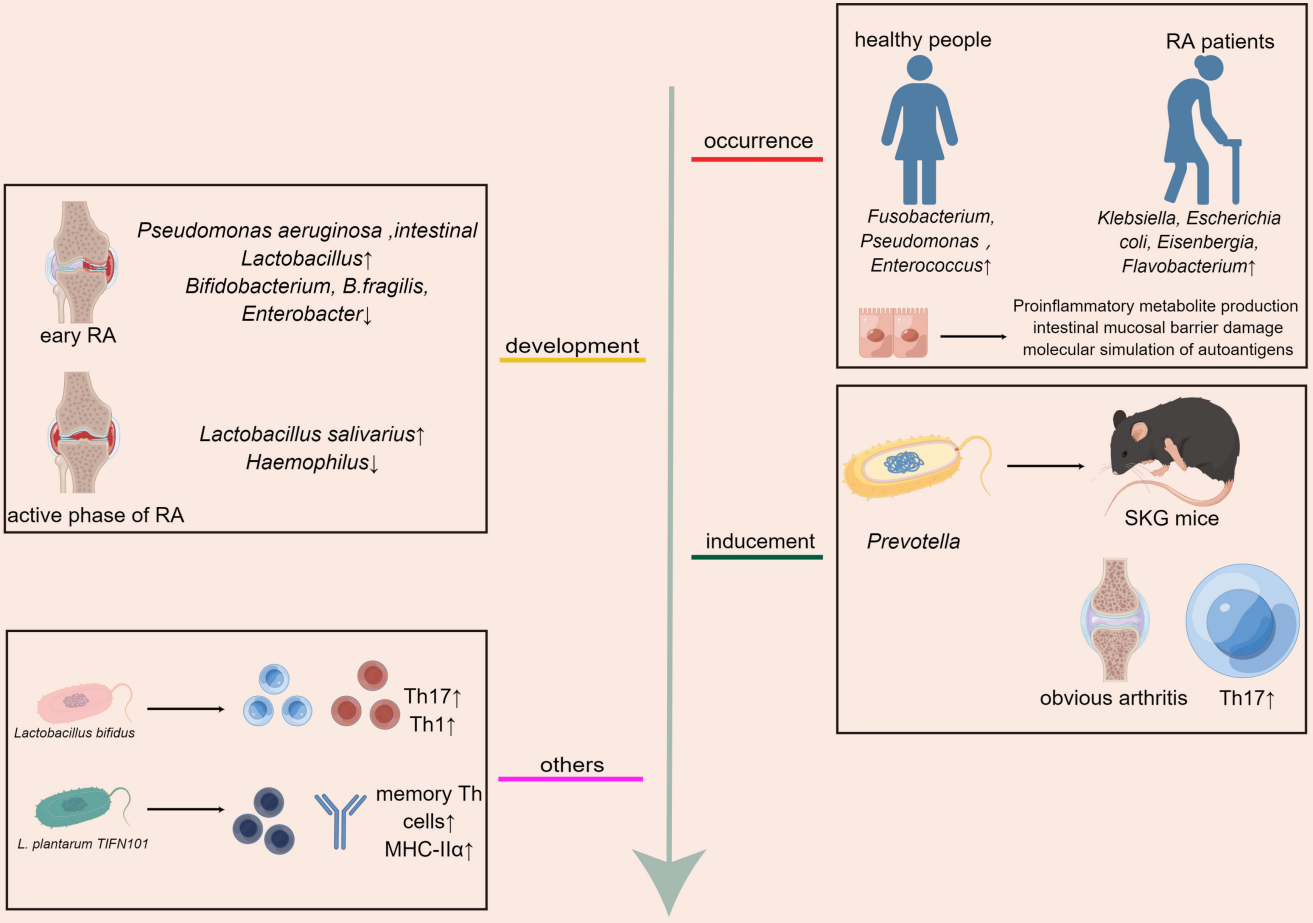
Gut microbiota affects the occurrence, development, and treatment of RA,the inflammatory responses of some species of microbiota may be also one of the mechanisms influencing RA pathogenesis.

Studies demonstrate that gut microbiota and its metabolites play a crucial role in the development of RA. First, the imbalance of gut microbiota can lead to the occurrence of RA. The comparative analysis of feces between RA patients and healthy people showed that the contents of *Klebsiella*, *Escherichia coli*, *Eisenbergia*, and *Flavobacterium* were higher in RA patients, and the contents of *Fusobacterium*, *Pseudomonas*, and *Enterococcus* were higher in healthy people (41). Roles of the gut microbiota in the pathogenesis of RA were also discussed by many studies and reviews through mechanisms including mainly production of proinflammatory metabolites, impairment of the intestinal mucosal barrier, and molecular mimicry of autoantigens (42). Furthermore, Inflammatory responses of some species of microbiota may be also one of the mechanisms influencing RA pathogenesis. *Lactobacillus bifidus* have showed effects on increasing the numbers of IL-17^+^ Th17 cells and activating Th1 cell responses which exacerbate RA (43). *L. plantarum TIFN101* which has effects on mucosal gene transcription, enhanced the intestinal mucosa immunity by increasing percentage of IL-17-producing activated memory Th cells and upregulated MHC-IIα (44).Moreover, the gut microbiota affects the development of RA. In patients with early RA, the abundance of *Pseudomonas aeruginosa* and *intestinal Lactobacillus* increased, *whereas* the fecal microbiota contained *Bifidobacterium*, *B.fragilis*, and *Enterobacter* decreased (42). In the active phase of RA, *Haemophilus* was depleted and the number of *Lactobacillus salivarius* increased (3). *Pseudomonas aeruginosa*, *Haemophilus*, and *Lactobacillus salivarius* are harmful bacteria, which can lead to metabolic disorders and destroy intestinal immunity (45). Intestinal *Lactobacillus*, *Lactobacillus acidophilus*, *Bifidobacterium, and Bacteroides fragilis* are beneficial bacteria with immunomodulatory effects and are closely associated with human health (46–48). In addition, the inducement of RA can be affected by adjusting gut microbiota. When SKG mice (RA model mice) were inoculated with the dominant flora *Prevotella*, the mice showed obvious arthritis, while when the mice were raised under sterile conditions or treated with antibiotics, no arthritis occurred, and the quantity of Th17 cells in the intestine of mice increased after fungal treatment (49) (**Figure 1**).

## 5 The mechanism of Traditional Chinese Medicine in preventing and treating Rheumatoid Arthritis by regulating Gut Microbiota

**Figure 2:**
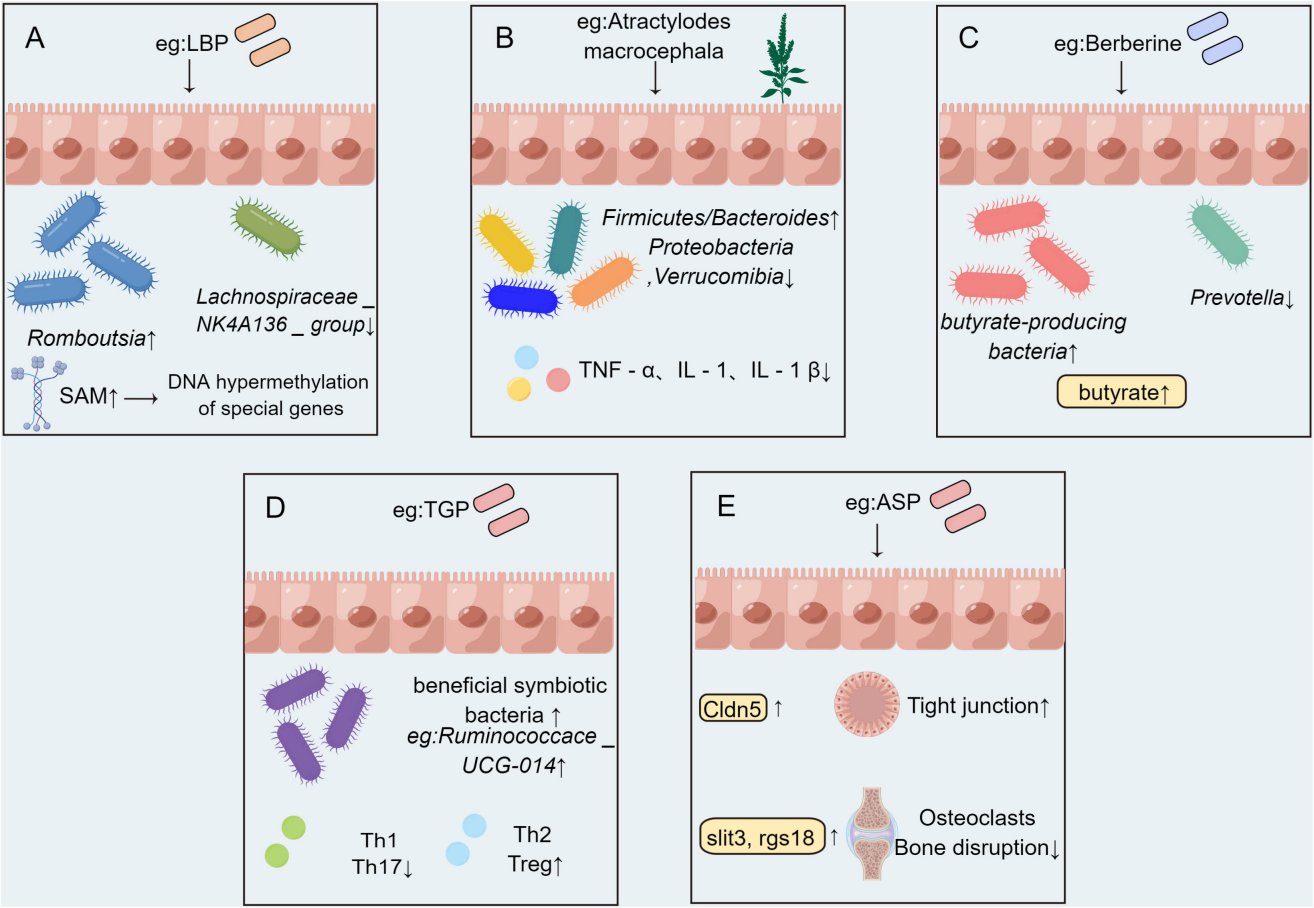
The possible mechanism of TCM affecting RA by acting on gut microbiota. **(A)** DNA hypermethylation of host intestinal epithelial related genes induced by Lycium barbarum polysaccharide (LBP) is related to the increase of bacterial metabolite S-adenosylmethionine (SAM) content. **(B)** Atractylodes koreana (Nakai) Kitam regulates gut microbiota and down-regulating the level of inflammatory factors. **(C)** Berberine can increase the abundance of butyric acid-producing bacteria and significantly increase intestinal butyric acid levels. **(D)** Paeonia glycosides (TGP) administration affected the structure of gut microbiota and down-regulated the levels of Th1 cells and Th17 cells, and up-regulated the levels of Th2 cells and Treg cells. **(E)** Angelica sinensis polysaccharide (ASP) shapes the composition of gut microbiota and regulates the expression of intestinal Cldn5, slit3 and rgs18.

TCM treatment can alleviate the symptoms of RA in a variety of ways. Gut microbiota is the largest source of microorganisms, and also the place where microorganisms interact with the human body (50). Dysregulated body state can lead to the imbalance of microbial composition and colonization and other functions, which in turn leads to the occurrence of autoimmune diseases (51). The effect of TCM on RA by acting on gut microbiota is divided into the following aspects. Firstly, the epigenetic modification of related genes could be modulated. After the intervention of Lycium barbarum polysaccharide (LBP), the abundance of *Lachnospiraceae _ NK4A136 _ group* and *uncultured _ bacterium _ f _ Ruminococcaceae* in CIA rat model decreased, and the abundance of *Romboutsia*, *Lactobacillus*, *Dubosiella*, and *Faecalibaculum* increased. LBP can increase the content of S-adenosylmethionine (SAM). The DNA hypermethylation of RA-related genes such as Dpep3 and Gstm6 in the host intestinal epithelium may be caused by increased SAM content (52). Secondly, proinflammatory cytokines were inhibited. Intestinal microflora disorders can trigger the abnormal activation of intestinal innate immune cells, leading to the up-regulation of pro-inflammatory cytokines and the reduction of anti-inflammatory cytokines (42).Atractylodes koreana (Nakai) Kitam can down-regulate inflammatory cytokines by regulating gut microbiota. After the treatment of CIA rats with Atractylodes koreana (Nakai) Kitam, the ratio of *Firmicutes/Bacteroides* increased and *Proteobacteria* and *Verrucomibia* decreased. At the same time,the inflammatory factors like TNF-α, IL-1, and IL-1β in the plasma of CIA rats decreased markedly, indicating that Atractylodes koreana (Nakai) Kitam can inhibit the generation of inflammatory cytokines in CIA rats and play a therapeutic role (53). Moreover, the abundance of specific flora increased in Complete Freund’s adjuvant (CFA) rats after LBP intervention. In rats, LBP intervention inhibited the pro-inflammatory cytokines IL-1α, IL-1β, TNF-α, and IL-6, thereby alleviating RA (54). Thirdly, the amino acid disorder could be improved. SCFAs can regulate intestinal endocrine function and play a significant role in host physiology (55). Berberine can reduce the diversity and abundance of intestinal bacteria in CIA rats but can increase the diversity of butyrate-producing bacteria, significantly increase the level of intestinal butyrate, and promote the production of butyrate by regulating gut microbiota as a therapeutic agent for RA (56). The therapeutic mechanism of improving synovial infiltration and vascular proliferation in RA rats after oral administration of Nakai Kitam may be related to the improvement of SCFAs imbalance in addition to the down-regulation of inflammatory factors (53). In addition to acting on SCFAs, TCM can also improve other amino acid metabolism through the gut microbiota. Wu-tou decoction (WTD) can partially inhibit inflammation and regulate gut barrier function by adjusting Bacteroides, Prevotella, Akkermansia and its related SCFAs, cholic acid, and indole propionic acid to improve RA (57)(**Figure 2**). Fourthly, T lymphocytes were intended to be regulated. Paeonia glycosides (TGP) intervention increased the relative abundance of beneficial symbiotic bacteria *Ruminococcace _ UCG-014*, *Oscillabacter*, and *Paraactoides* in CIA rats. In the meantime, TGP administration down-regulated the levels of Th1 cells and Th17 cells in CIA rats, and up-regulated the levels of Th2 cells and Treg cells. The effect of TGP on the dynamic changes of gut microbiota supports the hypothesis that the microbiota plays a role in the therapeutic effect of TGP-mediated CIA rats (58). Fifthly, the expression of related gene mRNA changed. Transcriptomics showed that Angelica sinensis polysaccharide (ASP) regulates Cldn5 to improve intestinal dysfunction induced by RA, regulates the expression of Slit3 and rgs18 to regulate the balance of osteoblasts and osteoclasts, which may be related to gut microbiota (59). Taken together, TCM has the characteristics of multi-pathway and multi-target in the treatment of RA, and RA is treated through a variety of pathways (12).

## 6 Conclusion

The interaction between RA and gut microbiota is mutual. RA can result in changes in gut microbiota, and gut microbiota affect the occurrence, development, and inducement of RA. TCM improves the structure and proportion of gut microbiota and regulates metabolism. Gut microbiota affects the absorption and metabolism of TCM, thereby improving efficacy, reducing toxicity, or producing toxic metabolites. TCM can treat RA by improving gut microbiota structure, adjusting T lymphocytes in the intestine, regulating microbiota metabolites, affecting intestinal immunity and intestinal barrier function, and improving intestinal dysfunction. TCM not only increases the therapeutic effect of conventional treatments but also reduces the side effects of therapeutic drugs.

Early RA usually begins several years to several months before obvious polyarthritis (60). With the continuous development of RA, inflammation aggravates, and organ dysfunction gradually leads to disability. Inhibiting the production of inflammation is the key point for the treatment of RA. However, the TCM to improve the gut microbiota in the treatment of RA has many problems. Firstly, the composition and dosage form of TCM are complex. The ingredients of TCM are complicated, and there are many monomer ingredients. After compatibility, some new effects will be produced, and it is difficult to explain the mechanism through monomer components. Moreover, TCM has different dosage forms, such as pill, powder, and decoction. Therefore, the oral absorption rate could be different, which will have different effects after gut microbiota absorption and metabolism. Secondly, the gut microbiota is complex and affected by many factors. The species and quantity of gut microbiota are numerous, and the dominant species are different at different periods of human growth and development. The composition of gut microbiota is also related to individual physique and state, so it is difficult to unify the conclusion. The gut microbiota is influenced by multi-factor and is numerous, and it is difficult to capture subtle changes.

The treatment of RA through gut microbiota has a broad application prospect, such as the current “bugs as drugs” bacterial-fecal transplantation therapy. The advantage of this therapy lies in the diversity of microorganisms, including not only bacteria but also viruses, fungi, etc. However, diversity and complexity also limit the reproducibility and measurability of microflora. Therefore, future research should focus on the development of specific microbial combination drugs with standard guarantees in drug purity, identity, and titer, to provide better measurability than fecal transplantation therapy. The beneficial mechanism of TCM in the treatment of RA by regulating gut microbiota is still at the preliminary stage of speculation. Future studies of TCM in the treatment of RA through gut microbiota can be carried out by establishing organoids, that is, by establishing a system highly physiologically related to the intestine of RA patients, to understand the effect of TCM on gut microbiota and the influence on inflammatory cytokines or inflammatory pathways in the intestine. On this basis, it can be verified by the knockout of related genes or the establishment of related transgenic animal models. In addition, after defining the specific gut microbiota, a comparative study of the microbiota can be performed to elucidate the changes in gut microbiota at low, medium, and high TCM doses. By observing the changes in specific gut microbiota structure anterior-posterior treatment with TCM, the structure of gut microbiota can be edited to clarify the pathogenic structure and treatment structure. In clinical research, large-scale sequencing of RA patients can be performed to establish a multi-center study to clarify the influence of TCM on gut microbiota in different RA patients with different ages, stages, and regions. More and more TCM and its active ingredients are needed to be identified and confirmed. TCM treatment of RA by intervening gut microbiota deserves further study.

## Author contributions

WW contributed to the conception of the study. YL wrote the first draft of the manuscript. ML and YC performed the literature research. WW and XW supervised the work and revised the manuscript. All authors contributed to the article and approved the submitted version.
