# Supplementary material for: Prevention and treatment of rheumatoid arthritis through traditional Chinese medicine: role of the gut microbiota
Source: Front Immunol. 2023 Sep 14;14:1233994. doi: 10.3389/fimmu.2023.1233994 (PMC10538529; doi:10.3389/fimmu.2023.1233994)
Supplement: Supplementary file 1 [file Table_1.pdf]

| component | Experimental subject | model | Effect on gut microbiota | Reference |
|---|---|---|---|---|
| lycium burbarum polysaccharide（LBP） | Rat | CIA | *Romboutsia, Lactobacillus, Dubosiella,Faecalibaculum*↑ *Lachnospiraceae_NK4A136_group, uncultured_ bacterum_f_Ruminococceae* ↓ | (54, 56) |
| berberine | Rat | CIA | butyrate-producing bacteria↑ *Prevotella*↓ | (58) |
| Total glucosides of paeony | Rat | CIA | beneficial symbiotic bacteria↑ | (61) |
| Polysaccharides and glycosides from Aralia echinocandins | Rat | CIA | *Roteobacteria, Acidobacteria ,Gemmatimonadetes*↑ *Tenericules,Fuso bacteria,Kiritimatiellaeota,Patescobacteria*↓ | (64) |
| Total Clematis triterpenoid saponins | Rat | CIA | *Firmicutes, Actinobacteria phyla*↑ *Bacteroidetes*↓ | (65) |
| Pterostilbene | Rat | Complete Freund's adjuvant (CFA) arthritis | *Helicobacter, Desulfovibrio, Lachnospiraceae,Mucispirillium*↓ | (66) |
| Angelica sinensis polysaccharide | Rat | CIA | *Lactobacillus, norank_f__norank_o__Clostridia_UCG-014,*etc↑ *Norank_f__Oscillospir-aceae, Roseburia*↓ | (67) |
| Clematidis Radix | Rat | CIA | *Firmicutes*↑ *Bacteroidetes*↓ | (68) |

| | | | | |
|---|---|---|---|---|
| Arsenic trioxide | Mouse | CIA | *Bacteroidetes*↑<br>*Firmicutes* ↓ | (69) |
| Atractylodes koreana (Nakai) Kitam | Rat | CFA | *Firmicutes/Bacteroides*↑<br>*Proteobacteria , Verrucomibia*↓ | (55) |
| Paederia scandens extract | Mouse | CIA | *Desulfovibrio, Mucispirillum, Helicobacter, Lachnospiraceae*↓ | (70) |
| Wu-tou decoction | Rat | Adjuvant arthritis (AIA) | *Lactobacillus*↑<br>*Bacteroides, Prevotella, Akkermansia*↓ | (60) |
| Danggui Niantong Granules | Rat | Adjuvant arthritis (AA) | *Firmicutes, Lachnospiraceae*↑<br>*Muribaculaceae, Bacteroidetes*↓ | (71) |
| Danggui Sini decoction | Rat | CIA | *g_Bacteroides*↑<br>*g_Bacteroides, g_Oscillibacter,etc*↓ | (39) |
| Ershiwuwei Lvxue Pill | Rat | CIA | *Genus Lactobacillus*↑<br>*Dorea,[Eubacterium]_ventriosum_group*,etc↓ | (72) |
| Twenty-Five Wei'er Tea Pills | Rat | CIA | Regulate metabolic function by *Lactobacillus*, *Bacteroides*, etc. | (73) |
| Zushima Tablet | Rat | CIA | Relieve disorders such as *phylum Bacteroidetes* and *Tenericutes* etc | (16) |
| Wantong Jingu Tablet | Rat | CIA | *Firmicutes / Bacteroidetes*↓<br>*Bacteroidetes, Tenericutes*, etc↓ | (74) |
| Huayu-Qiangshen-Tongbi formula | patients | RA | *Roseburiaw inulinivorans, Turicibacter sanguinis*,ect↑<br>*symbiosum and Clostridiales bacterium* 1-7-47FAA↓ | (17) |
| Yaobitong capsules | Rat | AIA | *Clostridia, Mollicutes*↑<br>*Erysipelotrichia, Bacteroides*↓ | (75) |
| Xiong Fu Powder | Rat | CIA | *Lactobacillus* species such as *L.acidophilus*↑ | (76) |

**Supplementary Table 1** TCM affects RA by regulating gut microbiota.

## References


16. Shan J, Peng L, Qian W, Xie T, Kang A, Gao B, et al. Integrated Serum and Fecal Metabolomics Study of Collagen-Induced Arthritis Rats and the Therapeutic Effects of the Zushima Tablet. *Front Pharmacol* (2018) 9:891. doi: 10.3389/fphar.2018.00891.
17. Mei L, Yang Z, Zhang X, Liu Z, Wang M, Wu X, et al. Sustained Drug Treatment Alters the Gut Microbiota in Rheumatoid Arthritis. *Front Immunol* (2021) 12:704089. doi: 10.3389/fimmu.2021.704089.
39. He Y, Cheng B, Guo B-J, Huang Z, Qin J-H, Wang Q-Y, et al. Metabonomics and 16s Rrna Gene Sequencing to Study the Therapeutic Mechanism of Danggui Sini Decoction on Collagen-Induced Rheumatoid Arthritis Rats with Cold Bi Syndrome. *J Pharm Biomed Anal* (2023) 222:115109. doi: 10.1016/j.jpba.2022.115109.
54. Lai W, Wang C, Lai R, Peng X, Luo J. Lycium Barbarum Polysaccharide Modulates Gut Microbiota to Alleviate Rheumatoid Arthritis in a Rat Model. *NPJ Sci Food* (2022) 6(1):34. doi: 10.1038/s41538-022-00149-z.
55. Pang J, Ma S, Xu X, Zhang B, Cai Q. Effects of Rhizome of Atractylodes Koreana (Nakai) Kitam on Intestinal Flora and Metabolites in Rats with Rheumatoid Arthritis. *J Ethnopharmacol* (2021) 281:114026. doi: 10.1016/j.jep.2021.114026.
56. Liu Y, Liu L, Luo J, Peng X. Metabolites from Specific Intestinal Bacteria in Vivo Fermenting Lycium Barbarum Polysaccharide Improve Collagenous Arthritis in Rats. *Int J Biol Macromol* (2023) 226:1455-67. doi: 10.1016/j.ijbiomac.2022.11.257.
58. Yue M, Tao Y, Fang Y, Lian X, Zhang Q, Xia Y, et al. The Gut Microbiota Modulator Berberine Ameliorates Collagen-Induced Arthritis in Rats by Facilitating the Generation of Butyrate and Adjusting the Intestinal Hypoxia and Nitrate Supply. *FASEB J* (2019) 33(11):12311-23. doi: 10.1096/fj.201900425RR.
60. Cheng X, Pi Z, Zheng Z, Liu S, Song F, Liu Z. Combined 16s Rrna Gene Sequencing and Metabolomics to Investigate the Protective Effects of Wu-Tou Decoction on Rheumatoid Arthritis in Rats. *J Chromatogr B Analyt Technol Biomed Life Sci* (2022) 1199:123249. doi: 10.1016/j.jchromb.2022.123249.
61. Peng J, Lu X, Xie K, Xu Y, He R, Guo L, et al. Dynamic Alterations in the Gut Microbiota of Collagen-Induced Arthritis Rats Following the Prolonged Administration of Total Glucosides of Paeony. *Front Cell Infect Microbiol* (2019) 9:204. doi: 10.3389/fcimb.2019.00204.
64. Li Y, Dai M, Wang L, Wang G. Polysaccharides and Glycosides from Aralia Echinocaulis Protect Rats from Arthritis by Modulating the Gut Microbiota Composition. *J Ethnopharmacol* (2021) 269:113749. doi: 10.1016/j.jep.2020.113749.
65. Guo L-X, Wang H-Y, Liu X-D, Zheng J-Y, Tang Q, Wang X-N, et al. Saponins from Clematis Mandshurica Rupr. Regulates Gut Microbiota and Its Metabolites During Alleviation of Collagen-Induced Arthritis in Rats. *Pharmacol Res* (2019) 149:104459. doi: 10.1016/j.phrs.2019.104459.
66. Rui Z, Zhang L, Li X, Han J, Yuan Y, Ding H, et al. Pterostilbene Exert an Anti-Arthritic Effect by Attenuating Inflammation, Oxidative Stress, and Alteration of Gut Microbiota. *J Food Biochem* (2022) 46(5):e14011. doi: 10.1111/jfbc.14011.
67. Hu Q, Wu C, Yu J, Luo J, Peng X. Angelica Sinensis Polysaccharide Improves Rheumatoid Arthritis by Modifying the Expression of Intestinal Cldn5, Slit3 and Rgs18 through Gut Microbiota. *Int J Biol*

*Macromol* (2022) 209(Pt A):153-61. doi: 10.1016/j.ijbiomac.2022.03.090.
68. Jiang S-Q, Pan T, Yu J-L, Zhang Y, Wang T, Li P, et al. Thermal and Wine Processing Enhanced Clematidis Radix Et Rhizoma Ameliorate Collagen Ⅱ Induced Rheumatoid Arthritis in Rats. *J Ethnopharmacol* (2022) 288:114993. doi: 10.1016/j.jep.2022.114993.
69. Niu S, Zhu X, Zhang J, Ma Y, Lang X, Luo L, et al. Arsenic Trioxide Modulates the Composition and Metabolic Function of the Gut Microbiota in a Mouse Model of Rheumatoid Arthritis. *Int Immunopharmacol* (2022) 111:109159. doi: 10.1016/j.intimp.2022.109159.
70. Xiao M, Fu X, Ni Y, Chen J, Jian S, Wang L, et al. Protective Effects of Paederia Scandens Extract on Rheumatoid Arthritis Mouse Model by Modulating Gut Microbiota. *J Ethnopharmacol* (2018) 226. doi: 10.1016/j.jep.2018.08.012.
71. Lu Q-J, Li J-Y, Lin H-X, Cai Y-S, Liu C-S, Fu L-P, et al. Danggui Niantong Granules Ameliorate Rheumatoid Arthritis by Regulating Intestinal Flora and Promoting Mitochondrial Apoptosis. *Pharm Biol* (2022) 60(1):1606-15. doi: 10.1080/13880209.2022.2107018.
72. Li Y, Liu C, Luo J, Zeng Y, Meng X, Wang S, et al. Ershiwuwei Lvxue Pill Alleviates Rheumatoid Arthritis by Different Pathways and Produces Changes in the Gut Microbiota. *Phytomedicine* (2022) 107:154462. doi: 10.1016/j.phymed.2022.154462.
73. Li Z, Nie L, Li Y, Yang L, Jin L, Du B, et al. Traditional Tibetan Medicine Twenty-Five Wei'er Tea Pills Ameliorate Rheumatoid Arthritis Based on Chemical Crosstalk between Gut Microbiota and the Host. *Front Pharmacol* (2022) 13:828920. doi: 10.3389/fphar.2022.828920.
74. Li Z-D, Qi F-Y, Li F. Integrating 16s Sequencing and Metabolomics Study on Anti-Rheumatic Mechanisms against Collagen-Induced Arthritis of Wantong Jingu Tablet. *Chin J Nat Med* (2022) 20(2):120-32. doi: 10.1016/S1875-5364(21)60080-8.
75. Shi W, Ye H, Deng Y, Chen S, Xiao W, Wang Z, et al. Yaobitong Capsules Reshape and Rebalance the Gut Microbiota and Metabolites of Arthritic Rats: An Integrated Study of Microbiome and Fecal Metabolomics Analysis. *J Chromatogr B Analyt Technol Biomed Life Sci* (2022) 1190:123096. doi: 10.1016/j.jchromb.2021.123096.
76. Xi X, Ye Q, Li X, Lu X, Fan D, Xia Y, et al. Xiong Fu Powder Regulates the Intestinal Microenvironment to Protect Bones against Destruction in Collagen-Induced Arthritis Rat Models. *Front Cell Infect Microbiol* (2022) 12:854940. doi: 10.3389/fcimb.2022.854940.
